# Effectiveness of Care for Child Development Program on the Sensitivity and Responsiveness Skills of Mothers

**DOI:** 10.22037/ijcn.v16i1.29797

**Published:** 2022-01-01

**Authors:** Ali BAHARI GHAREHGOZ, Seifollah HEIDARABADI, Hamid ALIZADEH, Mohammad ASGARI

**Affiliations:** 1 Psychology and Education of Exceptional Children, Department of Educational Sciences, Farhangian University,Tehran, Iran; 2 Department of Pediatric, School of Medicine, Tabriz University of Medical Sciences, Tabriz, Iran; 3 Faculty of Psychology & Education AllamehTabataba'i University, Tehran, Iran; 4 Assessment and Measurement Department, Faculty of Psychology, AllamehTabataba‘i University, Tehran, Iran.

**Keywords:** Sensitivity and Responsiveness, Mother-child Interaction, High-risk Infant, Intervention, Developmental Delay.

## Abstract

**Objectives:**

The present study was done to analyze the impact of the "care for child development" program on the sensitivity and responsiveness skills among mothers of children at risk of developmental delay.

**Materials & Methods:**

This study was a quasi-experimental research with a pretest-posttest design and experimental and control groups. The statistical population included all mothers with children at risk of developmental delay in Tabriz. Fifty mothers were selected through purposive sampling. Then, they were divided into two groups of 25 cases (one experimental group and one control group) using simple random sampling. The experimental group received training about the “care for child development“ program, while the control group received no training. The Social-Emotional Assessment/Evaluation Measure Family Profile (SEAM TM family profile) and Maternal Caregiving Quality Scale were the research measurement tools. The obtained data were evaluated by analysis of covariance (ANCOVA) and independent t-test using SPSS software version 20.

**Results:**

There was a significant difference between the experimental and control groups in maternal caregiving quality and responsiveness, provision of appropriate activities, predictable programs, and provision of play environment and safe home (P <0.05).

**Conclusion:**

The results showed that the care for child development program has positive effects on sensitivity and responsiveness skills of mothers of children at risk of developmental delays and can be considered and applied as a practical plan in national health policies.

## Introduction

The condition of child development under the age of 5 in Middle Eastern countries is worrying. In spite of a decline in the prevalence of developmental delay in most countries between 1990 and 2016, its frequency has increased significantly in some areas, such as North Africa and the Middle East (1). It is reported that the frequency of children exposed to developmental delay in Qazvin - Iran is 22.4% )^2^ (. Nowadays, considering the role of the environmental factors in the development of children, the field of child development moved from a medical model of exceptionality, in which it is assumed that a physical condition or disease exists in a patient, to an ecological model, where the child with exceptionalities has a complex interaction with many environmental forces (3). In the ecological model, due to the unique influence of the family system on child development, the significance of family in the formation of personality traits is increasing, and most early childhood interventions are directed to support the family environment. Hence, the child-centered intervention model has shifted to the family-centered intervention model (4). Moreover, family support is an integral part of the Individuals with Disabilities Education Act (IDEA), part C (3). Surveys of 4024 therapists from 60 countries showed that educating parents of children with developmental delays is an indispensable component of Part C of IDEA (5). 

Children are different from their birth. These differences mostly root in children’s genetic endowments. However, the emergence of their development and potentiality depends on environmental factors. These factors affect the development of children mostly through the family’s interaction with the child (6-8). 

 According to theoretical models, sensitivity and responsiveness comprise two of the important functions of the family (8). Responsiveness is an aspect of supportive parenting that plays an important role among different theories and research frameworks (such as attachment, social, and cultural theories) in establishing a robust structure for the desirable development of children. Responsive interactions occur when caregivers recognize the child's verbal and non-verbal signs and respond to them accordingly (8). Cordiality and parental responsiveness during infancy are prerequisites for the child's neurological, physical, and psychological development and have protective effects (9,10). Sensitivity and secure attachment between child and caregiver have positive effects on gray areas of the brain involved in social, cognitive, and emotional functioning (11). As the most positive effect on child development (8), they are also strong predictors of child development at the age of 5 (12). These positive effects are detectable both in short term and throughout the life of the child (13). Even responsive parenting has the potential to promote normal development for children at risk for developmental delay, such as premature children (14). In general, although the results of various studies have shown that responsive interactions and caregivers' sensitivity have a positive effect on the developmental skills of children under the age of 5 (15-21), the main question is what are the factors affecting responsive and sensitive interactions? Some researchers have shown that the ability of a caregiver to represent sensitive and responsive care is inherent and influenced by genetic and some environmental factors, such as external stressors (22). The existence of a premature infant may also influence this skill (23) and can be challenged by premature children. Since neonatal symptoms are not easily understood (24) and children's intelligence problems have a negative impact on maternal sensitivity (25), an increase in quantitative and qualitative knowledge of child caregivers has been emphasized (26, 27) because by educating and supporting child caregivers, it is possible to enhance the knowledge of parents regarding child-rearing, sensitivity, and responsiveness skills (28, 29).

Dunst and Kassow (2004) have identified different kinds of interventions that affect responsiveness and sensitivity of caregivers, including 1) Behavioral interventions aimed specifically at enhancing and promoting caregiver’s sensitivity (awareness, interpretation, responsiveness, etc.) to their children’s behavior, 2) providing social support (counseling, guidance, emotional reassurance, etc.) aimed at improving caregivers' self-esteem and competence, and (3) changing caregivers' understanding and awareness of their role in children's behavior (cognitive representation) (30). On the other hand, the results of some studies have shown that traditional models of education and support, which mainly include providing information to parents and teachers, did not meet the educational needs of caregivers, so that strategies of self-regulation (8), parent coaching (31) and self-directed learning (32) have been suggested to overcome the current challenges and accordingly, have designed various programs for child caregivers. One of these programs is the “care for child development” (CCD) program. This program is designed by the World Health Organization to empower caregivers, health workers, and individuals working with families to advance child development. With the support of coaching and to improve child development, many studies have acknowledged the effectiveness of this program in health services in some countries, such as Pakistan, India, and Turkey (33). Quantitative and qualitative indicators calculated by authors have shown that the proposed program had the proper validation features and could be considered as an applicable program in the health programs of Iran***.*** In this program, caregivers need three basic skills to make the optimum change in the child, including (1) sensitivity (being aware of the child's movements, gestures, and voice and interpreting them), (2) responsiveness: (responding in a timely manner and qualitatively to the child's signs and symptoms), and (3) learning enrichment and bonding (building trust and social relationships and establishing an emotional relationship between the child and the caregiver) (33). Some concerns are evident in the implementation of this program. Save the Children Organization, which has a strong commitment to implementing care for child development and pursuing it elsewhere, has considered the cost-effectiveness of interventions as one of the most important steps. Moreover, one of the key challenges of this program that impedes the development of efforts is the limited number of national coaches to guide training and executive activities (34). Finally, given the high prevalence of children at risk of developmental delay and in response to the above-mentioned concerns, this program has been modified by researchers to meet the needs of Iran. For the first time in Iran, its effectiveness on caregivers of children at risk of developmental delay was studied, and the researchers answered whether care for child development programs affects the level of sensitivity and responsiveness of caregivers of children at risk of developmental delay.

## Materials & Methods

This research was an applied study planned in quasi-experimental design with pre-test and post-test with 

Non-equivalent control group. The statistical population was all caregivers of children under the age of 3 at risk for developmental delay in Tabriz who were referred to educational and health centers in Tabriz during 2018-2019 selected by purposive sampling. Accordingly, 50 mothers of children, under the age of 3, at risk of developmental delay referring to Tabriz Child Development Center were selected and divided randomly into the experimental and control groups. A high-risk child is a Child with the age range of 4 to 36 months diagnosed by the following professionals: developmental pediatrician, pediatrics neurologist, neonatologist, and child and adolescent psychiatrist or based on the High-Risk Infants Program of Child Health Office of the Ministry of Health of Iran. 

**Inclusion criteria:**

1. Caregivers’ willingness to participate in the study;

2. The child’s age range of between 4 and 36 months;

3. No recognized neurodevelopmental disorders or delay, including autism spectrum disorder, cerebral palsy, and visual and auditory impairment;

 4. The performance of the child in at least one domain of the Ages and Stages Questionnaire be less than one standard deviation (between -1 and -2 SD) or cases diagnosed as a high-risk child by the following professionals: developmental pediatrician, pediatrics neurologist, neonatologist, and child and adolescent psychiatrist or on the basis of High-Risk Infants Program of Child Health Office of the Ministry of Health of Iran.

The exclusion criterion was the parents’ reluctance to participate in the study at any phase of the study. The independent variable in this study was the care for child development program, which was adopted by the researchers based on the response to the intervention approach. The experimental group was exposed to this variable, which was provided by their health and medical services and the control group received no conventional treatment. Also, during the intervention, none of the subjects were deprived of the existing standard interventions. Both groups were evaluated at the beginning of the study and three months after the study. Four sessions of two hours that were held weekly with two models of coaching and self-directed learning were considered (Table 2). Each session began with a challenging topic followed by caregivers discussing the problem and practicing the subject and skill in the classroom. The discussed subject was practiced for one week in the child's living environment by caregivers using self-monitoring tools (s/he was supposed to evaluate his/her performance in the process).

 The dependent variable was mothers' sensitivity and responsiveness skills to children at risk of developmental delay under the age of three. Data were collected using the Social-Emotional Assessment/Evaluation Measure (SEAM) family profile and Maternal Caregiving Quality Scale. The first tool examined the adequacy of caregivers in four areas related to the development of social-emotional skills. The questionnaire consisted of four criteria: 1) responding to the needs of the child, 2) providing activities that match the child's developmental level, 3) providing predictable schedule/routines for the child, and 4) providing a safe play and home environment for the child. Each criterion had 3-8 items. Caregivers indicated their competence in each item with four options of most often (score 3), sometimes (score 2), not yet (score 1), and not sure (score zero). Previous studies have shown that this scale has acceptable convergence with the social-emotional domain of the Ages and Stages Questionnaire, second edition (ASQ: SE-2) and the short form of Parenting Stress Index, Fourth Edition (PSI-4) (35). The psychometric properties of SEAM have not been evaluated in Iran. Researchers evaluated its content validity with the Iranian Expert opinions. The internal consistency of the scores of subscales was evaluated in the present study .

Maternal Caregiving Quality Scale has 32 questions to measure the quality of maternal care in terms of conflict and confusion, sensitivity and responsiveness, and availability. It is scored based on a five-point Likert scale (1 to 5). All factors had a high internal consistency (0.82 to 0.84), and the test-retest reliability of the scale indicated consistency of scores over a 20-day period (36). In this study, the sensitivity and responsiveness dimensions of this scale were used.

**Table 1:** Demographic characteristics of caregivers

Variable	levels	Experimental Group		Control Group		Total	
		Number	Percent	Number	Percent	Number	Percent
Care giver’s age (year)	<30	5	20	6	24	11	22
	30-40	14	56	15	60	29	58
	>40	6	24	4	16	10	20
Mothers’ level of education	Elementary school	5	20	2	8	7	14
	High school	6	24	9	36	15	30
	Bachelor and higher	14	56	14	56	28	56
Fathers’ level of education	Elementary school	7	28	3	12	10	20
	High school	2	12	9	36	12	24
	Bachelor and higher	15	60	14	52	29	56
						
						

**Table 2:** Educational content of the care for child development program

**session**	**Educational and Exercise content for each session**
**first**	Introduction to the program – Concept of development and its aspects - Factors affecting child development - Developmental delay - Child development monitoring
**second**	Who is a caregiver and how the child development can be cared - Fundamental skills for care and enhancing of the child’s development (sensitivity – responsiveness, and enrichment of the environment)
**third**	Methods of stimulating and enhancing of child development - Concept and significance of communication - Communication methods - Importance of playing – Supporting strategies for child play by a caregiver – toys - Types of play for children
**fourth**	Play recommendation activities and communication Based on six age groups - Identifying obstacles and solutions of conducting the program - Final monitoring of the development.

## Results

In this study, the level of education of 56 % of mothers and 56 % of fathers were bachelor or higher (Table 1). Table 3 indicates the mean scores of caregiving quality of child development based on the Social-Emotional Assessment/Evaluation Measure (SEAM) Family Profile and Maternal Quality Scale in the control and experimental groups in two stages of pre-test and post-test. The mean score of caregiver’s skills in the experimental group increased in the post-test.

**Table 3:** Descriptive characteristics (mean and standard deviation) of caregiving quality of child development

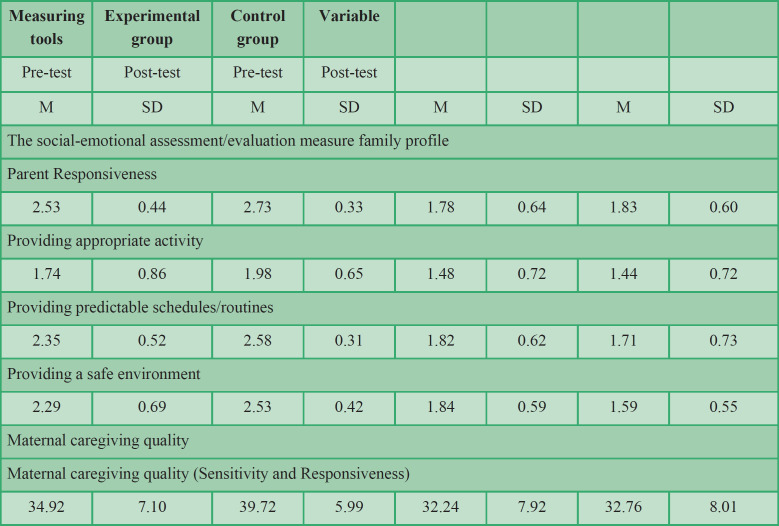

The ANCOVA was used to answer the research questions. First, the assumptions of the test, such as data normality, homogeneity of regression coefficients, and variance equality were examined. Due to the small sample size, the Shapiro-Wilk test was applied. The variables had a normal distribution at the confidence level of 0.95. Levene's test for the similarity of the two groups' variance was not significant for all variables except for the provision of appropriate activity. This defect is insignificant in this variable because ANCOVA is resistant to the assumption of homogeneity of variances when equating the number of subjects in groups (36). The assumption of homogeneity of the slope of regression was observed for maternal caregiving quality (F = 1.05, P> 0.05) and responsiveness dimensions (F = 3.7, P> 0.050). The regression slope homogeneity was not observed for appropriate activity provision (F = 6.2, P <0.050), predictability (F = 12.3, P <0.050), and provision of play and safe home environment dimensions (F = 9.9, P <0.050). Due to the non-homogeneity of regression slope assumption, instead of ANCOVA, an independent t-test was used for the provision of an appropriate activity, predictable programs, and provision of safe play and home environment dimensions.

According to the results of the presumptions, the ANCOVA was applied to compare the mean scores of post-test maternal caregiving quality and responsiveness dimensions after monitoring the pre-test in the two groups (Table 4). The result of univariate ANCOVA indicated that the effect of the group was significant (F = 14.57, P <0.05 and F = 10.88, P <0.05). After controlling pre-test scores, there was a significant difference between the mean scores of the post-test in the experimental and control groups regarding maternal caregiving quality and responsiveness, which is indicative of the effectiveness of care for the child development program on maternal caregiving quality and responsiveness. The Eta coefficient also indicated that 18% of the variations in maternal caregiving quality and 23% of the variations in the responsiveness of the variable can be explained by the care for child development program.

**Table 4:** Results of univariate ANCOVA of the effect of care for the child development program on maternal caregiving quality and responsiveness of caregivers dimensions

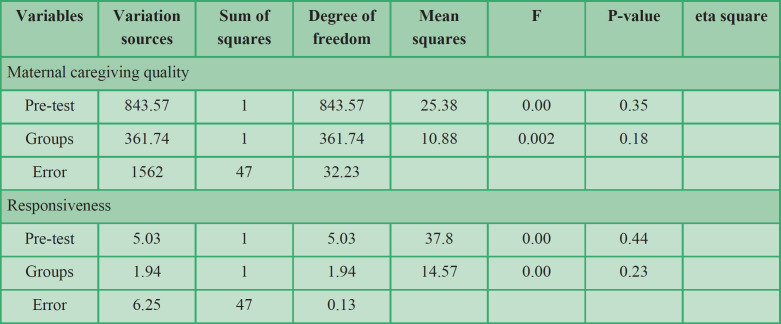

The mean difference of provision of appropriate activities, predictable programs, and provision of a safe environment are shown in Table 5. The mean difference between groups regarding provision of appropriate activities (t = 2.02, P <0.050), predictable programs (t = 2.3, P <0.050), and provision of a safe home and play environment (t = 3.8, P <0.050) was statistically significant. In other words, the care for child development program was effective in promoting the competency of caregivers in all three variables.

**Table 5:** Results of the mean difference of the variables in the experimental and control groups

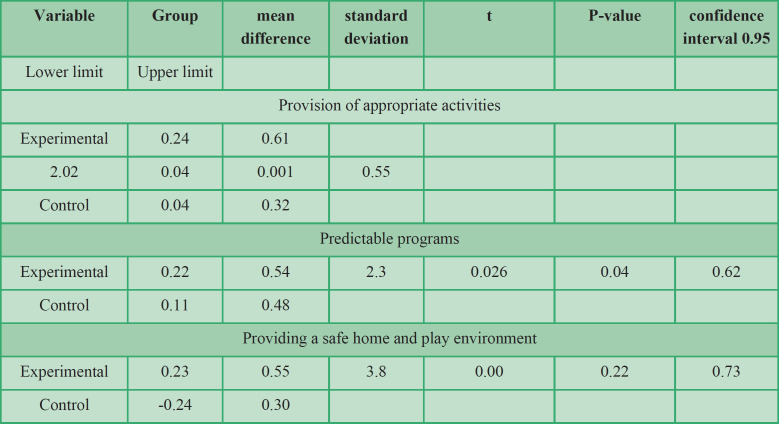

## Discussion

The present research was done to study the effect of the CCD program on the quality of maternal caregiving quality and maternal responsiveness of children at risk of developmental delay. The results of the ANCOVA acknowledged the effectiveness of the CCD program on maternal caregiving quality and responsiveness. The Eta coefficient indicated that 18% of the variations in maternal caregiving quality and 23% of the variations in the responsiveness of the variable can be explained by the CCD program. In addition, the results of comparing the mean difference between the two experimental and control groups showed that the CCD program was effective in promoting the competency of caregivers in all three variables of providing appropriate activities, predictable programs, and provision of play environment and safe home.

The results of this study indicated that the CCD program was effective in empowering caregivers and enhancing maternal caregiving quality and their responsiveness, which is in line with the results of research by Evans et al. (20), Dunst and Kassow (30), and Lavallee et al. (40), showing that despite the inheritance nature of sensitivity and responsiveness skills and the potential for them to decline due to the risk of the child for developmental delay (22), maternal caregiving quality and caregivers’ responsiveness can be improved by CCD program.

Also, the CCD program was effective for caregivers’ sense of adequacy in providing a safe play and home environment, which is in line with the research by Feldman and Case on the effectiveness of educating skills of parenting and child safety (39). This program emphasizes the role of a clean, safe, and protected environment in preventing injury for discovery and learning new skills (40d T provided caregivers with the skills required for safe environment provision.

We also found that the CCD program was effective in promoting the competency of caregivers in providing appropriate activities. The program emphasizes the provision of appropriate activities by caregivers based on their age and type of problem using a counseling card. Caregivers select and execute child-related activities, communication activities, or games appropriate to the age or type of child's problem (40). Educating this issue provided the caregivers with the skills needed in providing appropriate activities.

Another result of this study was that the CCD program was effective in promoting the competency of caregivers in the provision of predictable plans. An important focus of the program is to highlight the availability, consistency, and predictability of caregivers in several daily activities, such as eating and sleeping, and combining play activities and communicating with daily activities so that caregivers are more confident about the conditions necessary to stimulate curiosity and learning around child surroundings (40).

Overall, the results of this study specified that support and educational programs for parents with children at risk for developmental delay have positive effects on parents, which can be explained by different aspects of the effectiveness of this program. In terms of the approach of interventions, this program is a combination of behavioral interventions, social support, and cognitive representations, which is emphasized by Dunst and Kassow for enhancing the sensitivity and responsiveness skills of parents (30). The combination of different interventions has probably increased the program’s effectiveness. From the perspective of behaviorist theories, it is likely that helping parents understand their child's unique characteristics and providing guidance on how to build a positive relationship and interaction with their children is rewarding and reinforcing for parents. In this program, the caregiver learns with the help of techniques of the "Look, Listen, and Touch" to understand the child's interests and feelings better and respond accordingly to the child's needs and interests. These techniques help parents to manage their children better and facilitate interaction between the caregivers and the child. Also, caregivers feel competent in sensitivity and responsiveness skills. On the other hand, as role-playing is a part of the CCD program, some caregivers learn about sensitivity and responsiveness through observational learning during the implementation of the program. It can also be explained from the perspective of a self-directed learning theory that focuses on adult learning. In this program, promoting and protecting the development of children at risk are considered an important goal for caregivers, and they have a relatively high motivation to achieve this goal. On the other hand, prior knowledge of caregivers, which they achieved through formal and informal resources coupled with this new knowledge, provides possible ways to move toward the primary goal of promoting child development. The caregivers will engage in learning activities in various situations, such as group activities in the classroom and individually at home. They evaluate their performance through self-monitoring tools as well as program orientation from coaching to training over time that reinforces a sense of competence, independence, and self-worth in caregivers, which include targeting and motivation, goal orientation, and performance activities (41).

## Conclusion

 Lack of control over some important variables, such as socioeconomic status and mental health status of caregivers, as well as data collection based on caregiver self-reporting, are limitations of this study. Due to the positive impact of the program on caregivers in the present research and other research from other countries and the simplicity of the program, and its non-dependence on special instruments, this program seems to be highly applicable and can easily be used at home, kindergartens, and health centers. It can also be used as an applicable program in the health system of countries.

## Author’s contribution

Ali Bahari Gharehgoz : Study concept and design, development of the original idea, collected and analyzed the data, interpretation of data

 Seifollah Heidarabady : thesis advisor , wrote the paper, and approved the final version to be published

Hamid Alizadeh: Thesis Supervisor, revising it critically for important intellectual content, Monitored intervention process ,

 Mohammad Asgari: thesis advisor , analyzed the data

All authors agreed to be accountable for all aspects of the work in ensuring that questions related to the accuracy or integrity of any part of the work are appropriately investigated and resolved. 

## Conflict of interest

The authors declare that there is no conflict of interests
